# Sol–Gel Derived Alumina Particles for the Reinforcement of Copper Films on Brass Substrates

**DOI:** 10.3390/gels10100648

**Published:** 2024-10-11

**Authors:** Samah Sasi Maoloud Mohamed, Marija M. Vuksanović, Dana G. Vasiljević-Radović, Ljiljana Janković Mandić, Radmila M. Jančić Heinneman, Aleksandar D. Marinković, Ivana O. Mladenović

**Affiliations:** 1 Faculty of Technology and Metallurgy, University of Belgrade, Karnegijeva 4, 11000 Belgrade, Serbia; samahsasi1@gmail.com (S.S.M.M.); radica@tmf.bg.ac.rs (R.M.J.H.); marinko@tmf.bg.ac.rs (A.D.M.); 2 VINČA Institute of Nuclear Sciences—National Institute of the Republic of Serbia, University of Belgrade, 11000 Belgrade, Serbia; ljmandic@vin.bg.ac.rs; 3 Institute of Chemistry, Technology and Metallurgy, University of Belgrade, Njegoševa 12, 11000 Belgrade, Serbia; dana@nanosys.ihtm.bg.ac.rs

**Keywords:** alumina, sol–gel, copper films, co-electrodeposition, microhardness, adhesion

## Abstract

The aim of this study is to provide tailored alumina particles suitable for reinforcing the metal matrix film. The sol–gel method was chosen to prepare particles of submicron size and to control crystal structure by calcination. In this study, copper-based metal matrix composite (MMC) films are developed on brass substrates with different electrodeposition times and alumina concentrations. Scanning electron microscopy (FE-SEM) with energy-dispersive spectroscopy (EDS), TEM, and X-ray diffraction (XRD) were used to characterize the reinforcing phase. The MMC Cu-Al_2_O_3_ films were synthesized electrochemically using the co-electrodeposition method. Microstructural and topographical analyses of pure (alumina-free) Cu films and the Cu films with incorporated Al_2_O_3_ particles were performed using FE-SEM/EDS and AFM, respectively. Hardness and adhesion resistance were investigated using the Vickers microindentation test and evaluated by applying the Chen–Gao (C-G) mathematical model. The sessile drop method was used for measuring contact angles for water. The microhardness and adhesion of the MMC Cu-Al_2_O_3_ films are improved when Al_2_O_3_ is added. The concentration of alumina particles in the electrolyte correlates with an increase in absolute film hardness in the way that 1.0 wt.% of alumina in electrolytes results in a 9.96% increase compared to the pure copper film, and the improvement is maximal in the film obtained from electrolytes containing 3.0 wt.% alumina giving the film 2.128 GPa, a 134% hardness value of that of the pure copper film. The surface roughness of the MMC film increased from 2.8 to 6.9 times compared to the Cu film without particles. The decrease in the water contact angle of Cu films with incorporated alumina particles relative to the pure Cu films was from 84.94° to 58.78°.

## 1. Introduction

Alumina and aluminum hydroxides are notable materials due to their structural and property characteristics, which can be precisely tailored through appropriate processing methods [1]. A variety of distinct structures can be selected [2] to achieve the desired particle morphology and size, enabling the material to exhibit properties designed to fit the requirements of the final application [3,4]. In certain low-temperature applications, surface modification, using silane and organic modifiers on the particles, can significantly improve their incorporation into a polymer matrix [5]. When the particles are prepared for use in the metal matrix, the organic molecules attached to the surface suffer during the preparation of the composite, and therefore, the chosen way to prepare suitable particles is to control their size and crystal structure [6,7]. The morphology of prepared particles could also influence the properties of the prepared films in several ways. The film hardness can be improved, the film can change surface energy, and adhesion to the substrate can be modified.

The sol–gel technique allows for an easy way to produce very fine alumina particles using relatively accessible laboratory conditions and to control the quality of the particles. This way of producing the particles enables the control of their chemical composition and tuning of their properties. The exact crystal structure of the material is controlled by the calcination process that follows the first step, maintaining the fine grain and crystallite size.

Copper is used as a material that has several good properties, such as an aesthetic one where the material is covered to have a specific colour and resistance to the environment. On the other hand, copper has some interesting properties in contact with some chemicals and environments [8] and can be used to carry the particles that could eventually exhibit some specific activity [9]. Copper itself has very low hardness and is not very resistant to wear and mechanically harsh environments. When it is meant to serve as the matrix of the composite, the intention is to obtain a material that is harder, stronger, and more resistant to environmental impact [9]. Also, if it is possible to control properties such as surface energy or water or oil affinity, it can change the possible use of this sort of material [10].

The incorporation of alumina particles into copper matrices via electrochemical methods has been extensively studied in recent decades, primarily using commercial particles [11,12,13]. The sol–gel technique involves using substances that are high in highly reactive chemicals as precursors. These precursors combine with solvents to produce reactions like alcoholysis and hydrolysis. A stable and transparent sol matrix forms within the solution as a result of these processes [14,15]. The use of sol–gel-prepared alumina to reinforce MMC films represents a novel approach. Additionally, the synergistic effects of film thickness and alumina concentration on the composite films’ absolute hardness [16] and adhesion parameters [17,18] have not been previously investigated. The effects of alumina are most often observed by varying the electrochemical parameters [11]. Thiemig et al. [12] studied the impact of the pulse plating (PC) parameters on the co-electrodeposited γ-Al_2_O_3_ particles in Cu- and Ni-based MMC films and compared them to DC (direct current), PC, and PRC (pulse reverse current) plating, whereby pulsed deposition modes are favoured due to greater incorporation of alumina into the composite. Further information on the co-deposition mechanisms of inert, semi-conductive, and conductive particles in copper films on metal substrates was provided by [13]; the study utilized α-Al_2_O_3_ nanoparticles (300 nm) and demonstrated that particle content in films increased with current density. Additionally, the effects of varying potential differences during the co-electrodeposition of Cu-Al_2_O_3_ composites were examined [19]. The authors used α-Al_2_O_3_ nano-sized alumina (≈40 nm) particles and indicated that alumina has not been deposited at lower magnitudes of potential differences. On the other hand, it is found that the surface wettability of Cu-Al_2_O_3_ composite films increases as the potential difference increases, and more hydrophilic films are obtained, and as such, they are suitable for the enhancement of heat transfer applications [19].

Numerous studies have explored the preparation of MMC films on material surfaces, making this a widely researched topic. The deposition conditions used in this study were previously optimized for copper films. Composite films are prepared using electrochemical deposition/co-deposition under uniform current density. Ceramic particles are maintained in suspension in electrolytes through continuous stirring and agitation. The size of the particles needs to be submicrometric. The thickness of the deposit is regulated by the duration of the deposition process [20] and verified by the mechanical comparator [18]. Materials produced this way should exhibit improved hardness, adhesion to the substrate, and water affinity, as the added particles are relatively polar. However, challenges include achieving uniform particle distribution both throughout the film’s depth and on its surface. Additionally, the particles may alter the film’s microstructure, affecting the shape and size of the forming crystals. Those properties are studied and correlated with other material properties. Roughness can be influenced by the properties of the reinforcement as well as by the conditions of film formation [16,21].

This paper will present the results of the preparation of alumina particles with the aim of obtaining them to be suitable for film deposition with appropriate properties, and these properties include surface hardness, surface wettability, and roughness. This study aimed to prepare Cu-Al_2_O_3_ nano-microscale composites, where alumina particles were synthesized via the sol–gel method and then co-deposited with the matrix using electrodeposition, and to study the effect of alumina on the film properties. The description of the synthesis of alumina nanoparticles according to sol–gel is given in Section 4.1. The study of grain refinement/non-refinement of copper and its composite, for the particles and phases present, in terms of roughness, wettability, hardness, and adhesion using different techniques, was applied. The alumina contents in the electrolyte were adjusted to be 0, 1.0, 3.0, and 5.0 wt.% in the sulphate electrolyte. Film thicknesses of 2, 22, and 52 μm were adjusted based on the deposition rate (electrodeposition time) for both cases for the deposited films without/with alumina. A comparative study with all the advantages and disadvantages of the synthesized material is presented to understand the possibility of applying different synthesis methods in a cheap, fast, economical, and eco-friendly way. The goal of this research is to enhance the composite film’s surface topography and mechanical characteristics over pure copper film with minimal degradation.

## 2. Results and Discussion

This investigation includes two research directions: (1) synthesis and characterization of alumina particles and (2) synthesis and characterization of copper films without/with alumina particles with a characterization of the metallic substrate prior to deposition of the films.

### 2.1. Microstructural and Chemical Characterization of Alumina Nanoparticles

#### 2.1.1. Microstructure of Al_2_O_3_-FE/SEM and TEM

**Figure 1:**
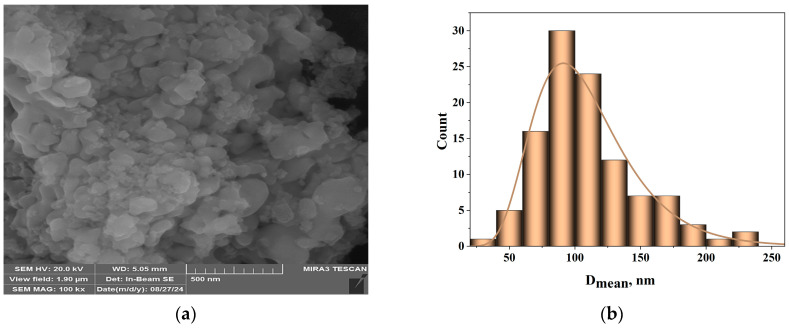

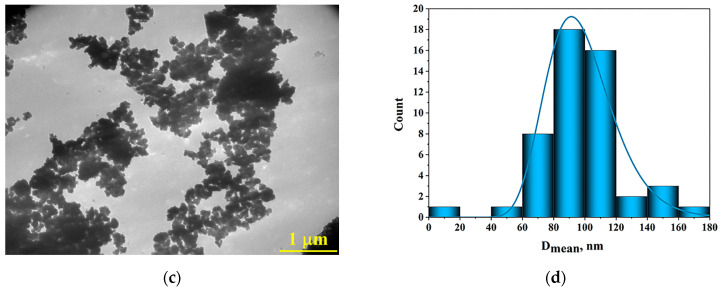
Particle morphology of alumina: (**a**) FE-SEM picture, (**b**) diameter size, (**c**) TEM micrographs, and (**d**) corresponding histogram of particles.

An FE-SEM microscope was used to examine the alumina particles prepared in the sol–gel process. The morphology of the particles is shown in Figure 1a. The mean diameter of the particles was determined using FE-SEM images and Image Pro Plus 6.0 image analysis software (Media Cybernetics, Rockville, MD, USA) [22]. Figure 1b displays the particle size distribution. The histogram that was obtained indicates that most of the particles had a diameter of around 100 nm.

Micrograms obtained by transmission electron microscopy (TEM) show an inhomogeneous distribution of different sizes of submicrometre agglomerates and nano-sized particles of alumina powder (Figure 1c). Spherical–oval-shaped particles were observed in individual locations where no agglomerates were formed and their size is 100–120 nm, which agrees with SEM analysis (Figure 1d).

#### 2.1.2. Chemical Purity of Al_2_O_3_-EDS/Mapping

**Figure 2:**
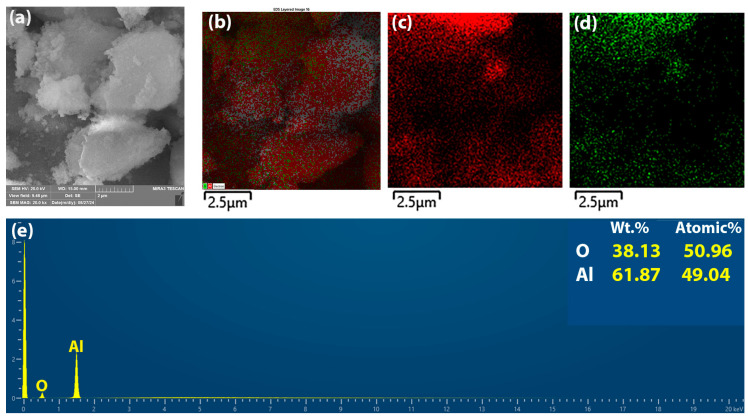
(**a**) SEM images of alumina particles, (**b**) elemental mapping, (**c**) Al, (**d**) O, and (**e**) EDS spectra.

The elemental composition of the generated alumina particles was evaluated using EDS analysis on the FE-SEM images (Figure 2). Based on the EDS analysis, there is an even distribution of Al and O atoms in the particles, confirming the successful synthesis of alumina particles from sol–gel. The EDS mapping did not show other elements such as chlorine that existed in precursors, so the synthesis method can be considered as appropriate for this purpose.

#### 2.1.3. XRD Analysis of Al_2_O_3_

**Figure 3:**
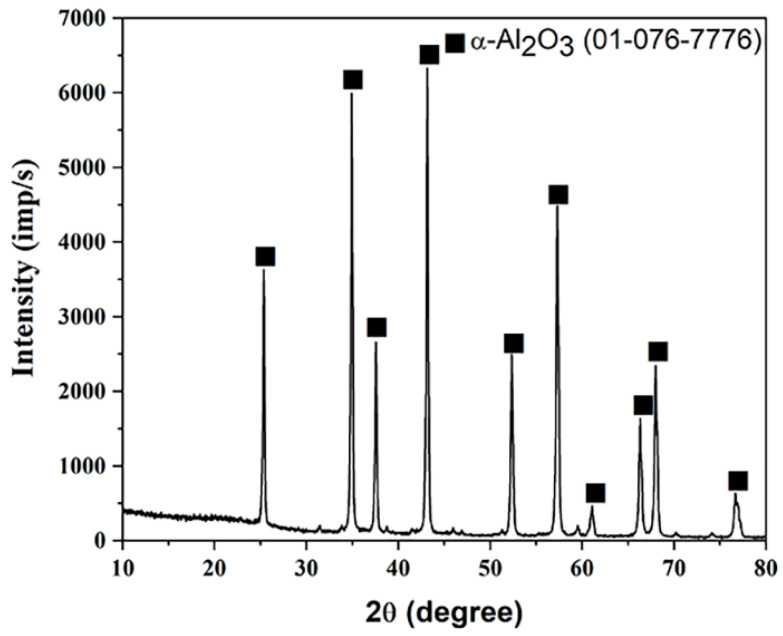
X-ray diffractogram of alumina powder obtained by sol–gel method.

An XRD spectrum of the sol–gel-derived nanoparticles of alumina powder is shown in Figure 3. All peaks were assigned by the standards No. 01-076-7776 (for α-Al_2_O_3_) [23].

The alumina with a rhombohedral structure that is most stable is called α-Alumina and is proven to be preset in the crystal structure. At 2θ values of 34.8°, 43.1°, and 57.3°, respectively, three significant distinctive peaks have been detected from the planes (104), (113), and (116) [23].

XRD patterns confirmed that most intensive reflections came from Al_2_O_3_ applying the pseudo-Voigt profile. Refined structural parameters include the lattice constant as well as the average crystallite size values obtained by applying the pseudo-Voigt profile. The values obtained for the lattice parameters were a_h_ = b_h_ = 4.7495 Å, c_h_ = 12.9718 Å, and *V* = 253.41 Å. The average crystallite size (*D*) was calculated based on the full width at the half-maximum intensity (FWHM) of the main reflections by applying Scherrer’s formula (see Supplementary Material A). The size of the alumina crystallites is 32.7 nm. The fractional coordinates for O and Al are given in Table 1, where x, y, and z are the fractional coordinates for O and Al.

### 2.2. Characterization of Substrate, Copper Films, and MMC of Cu-Al_2_O_3_ Films

An investigation of the effect of alumina nanoparticles as reinforcement on the morphology, roughness, microhardness, adhesion strength, and wettability of electrolytically produced Cu films was conducted through the examination of Cu films formed with varying deposition times and alumina concentrations in suspension. To better understand how this reinforcement affected the Cu film characteristics, they were compared with films formed from the same acidic basic sulphate electrolyte that did not contain alumina nanoparticles.

The first factor influencing the structural, mechanical, and topographical properties of Cu and Cu-Al_2_O_3_ films are the properties of the chosen metallic substrate and the possibility of modifying its topography in order to achieve the best adhesion of the films.

#### 2.2.1. Characterization of Substrate

##### Topography of Brass Substrate after Mechanical and Chemical Modification

**Figure 4:**
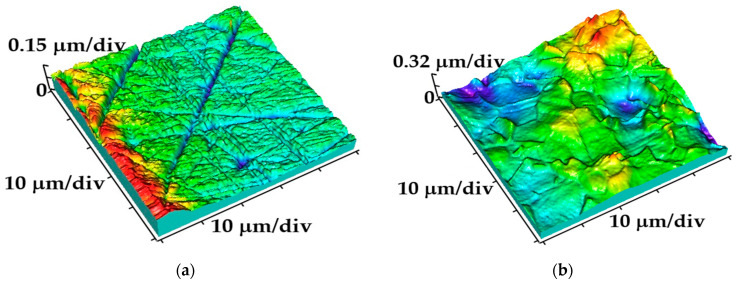
The three-dimensional (3D) images of the brass substrate obtained on an AFM device: (**a**) brass surface after mechanical grinding and (**b**) after chemical etchings in a mixture of acids (HNO_3_:H_3_PO_4_:CH_3_COOH = 4:11:5 vol.%) at 10 s.

The brass substrate was mechanically polished using SiC paper and the topography is presented in Figure 4a. Based on the roughness analysis, the average surface roughness parameter (*S*_a_) was found to be 31.30 nm. The next step included a chemically roughened sanded brass surface aimed to remove deep channels and ridges and obtain sharp peaks for a better contact surface for the electrodeposition process (Figure 4b). The obtained roughness parameter *S*_a_ is 80.43 nm. The red color indicates the maximum height of the grain, while the blue indicates the depression.

Any modification of the surface of the substrate leads to a change in the microstructure, and therefore, the mechanical properties of the substrate. Before applying the films to a substrate, it is necessary to evaluate the mechanical properties (microhardness) of the chemically modified substrate without the film.

##### Microhardness of Brass Substrate after Chemical Modification

**Figure 5:**
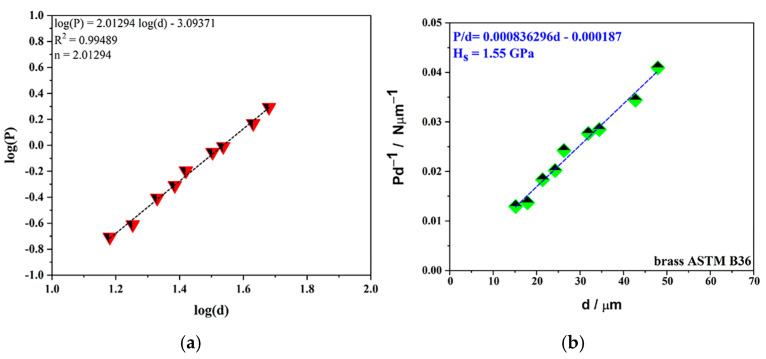
Mechanical properties of brass substrate after chemical etching: (**a**) calculation of Meyer’s index and evaluation ISE, and (**b**) calculation hardness of substrate with application PSR model.

Figure 5a shows the relation between the applied load, *P*, and the measured values of the Vickers microhardness diagonal size, *d*, in the log–log scale. The size and character of the ISE effect (Indentation Size Effect) for brass substrates after chemical etching can be evaluated in different ways (Meyer’s power law, Proportional Specimen Resistance (PSR), and Hays–Kendall methods) [24]. Meyer’s power law and index *n* or “work-hardening index” for the prepared brass substrate was 2.01294. The value obtained for *n* (index *n* > 2) indicates the existence of a reverse ISE (RISE) [24]. The Proportional Specimen Resistance (PSR) method [25,26,27,28] was chosen to determine the absolute hardness of the substrate (Figure 5b). The calculated value of the microhardness of the chemically etched brass substrate was 1.55 GPa.

#### 2.2.2. Characterization of Copper Films and Cu-Al_2_O_3_ MMC Films

##### Microstructure of Free Cu and Cu-Al_2_O_3_ Composite Films-FE/SEM

**Figure 6:**
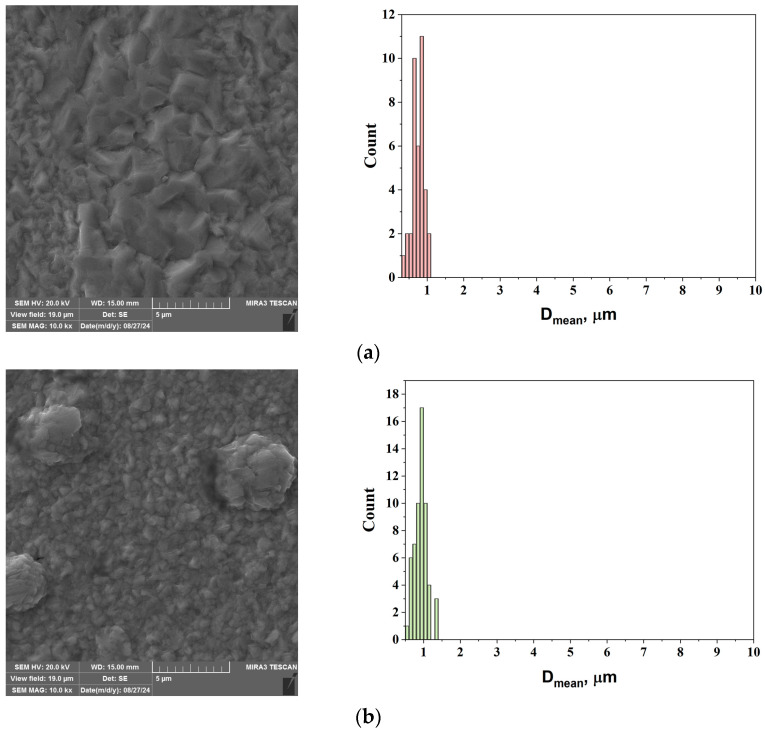

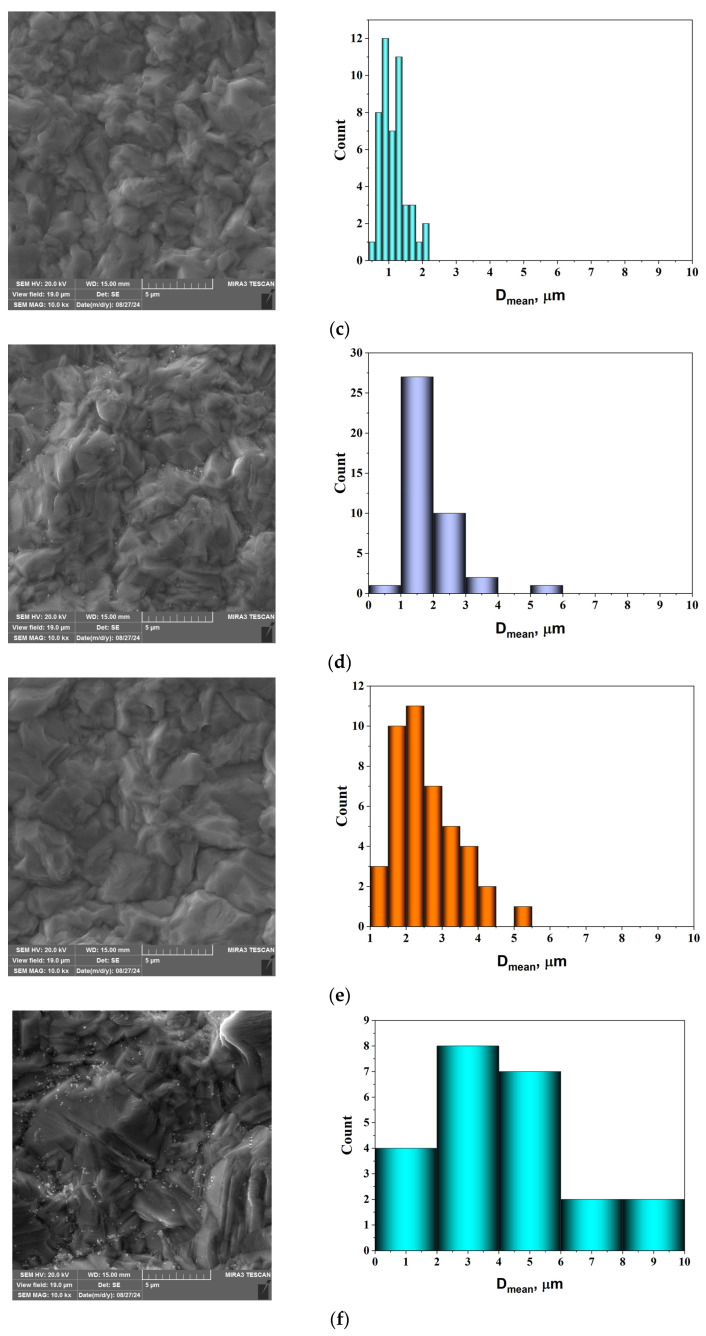
Surface morphology analyses of electrodeposited alumina-free Cu and MMC Cu-Al_2_O_3_ films electrodeposited/co-electrodeposited on the brass substrate with/without constant wt.% of alumina particles (1.0 wt.%) and with different thicknesses of the films: (**a**) 2 μm, Cu; (**b**) 2 μm, Cu-Al_2_O_3_; (**c**) 22 μm, Cu; (**d**) 22 μm, Cu-Al_2_O_3_; (**e**) 52 μm, Cu; and (**f**) 52 μm, Cu-Al_2_O_3_. The magnification was ×10,000 for all pictures.

Figure 6 shows the surface morphologies of the alumina-free copper films (Figure 6a,c,e) electrodeposited from ABSEs and Cu-Al_2_O_3_ MMC films electrodeposited from ABSE-Al-1% electrolytes (Figure 6b,d,f) with different film thicknesses (2, 22, and 52 μm). The electrodeposition mode was DC with a current density of 50 mA·cm^−2^. Figure 6 also shows the corresponding grain size distribution histograms generated from image analysis of the FE-SEM micrographs.

The presented morphologies of all films are very coarse. The grain size and grain distribution are different for the Cu-Al_2_O_3_ and the pure Cu films. For the same thickness of the films with/without incorporated alumina particles, no discernible morphological difference in grain type was observed. The presence of relatively large crystals was observed in the DC mode in both cases, without/with alumina. This behaviour is expected since there is no change in the electrochemical deposition parameters. The only change in the microstructure was observed in the thinnest sample, which can be attributed to the inhibitory effect of alumina on the growth of copper crystals (Figure 6b). The structural change in co-electrodeposited metallic films is described by the electro-crystallization process, in particular, the combined process of nucleation and crystal growth [29]. Each Cu film is microcrystalline (mc) and compact. The presence of nano-alumina particles on the Cu surface is only observed in thicker films (Figure 6d, f). Bright, spherical, and small alumina nanoparticles were co-deposited on the surface, mostly at the grain boundaries. The mean grain size (D_mean_) for each film produced was determined based on grain size distribution histograms, and Table 2 shows the clear grain boundary.

A 2 µm thick Cu film containing alumina nanoparticles has the smallest grain size (0.752 μm). As the deposition time increased, the grain size also increased, reaching a maximum film thickness of 4.094 µm for the 52 µm thick Cu-Al_2_O_3_ films (Table 2) when compared to samples co-electrodeposited from electrolyte ABSE-Al-1%. During the co-deposition of Cu and alumina particles, the increased concentration of alumina in the electrolyte interferes with the metal nucleation process by increasing the surface energy [30]. This leads to the growth of larger crystals and the appearance of a coarser deposit. The grain size of the Cu-Al_2_O_3_ film co-electrodeposited from electrolyte ABSE-Al-5% was 4.466 μm. This confirms that the dominant process is crystallization and that crystalline grain growth is favoured. The morphologies of the MMC films obtained from electrolytes ABSE-Al-3% and ABSE-Al-5% with the corresponding grain size distribution histograms are shown in the Supplementary Materials (Figure S1a,b).

##### Chemical Analysis of Metal Cu and MMC Films of Cu-Al_2_O_3_-EDS

**Figure 7:**
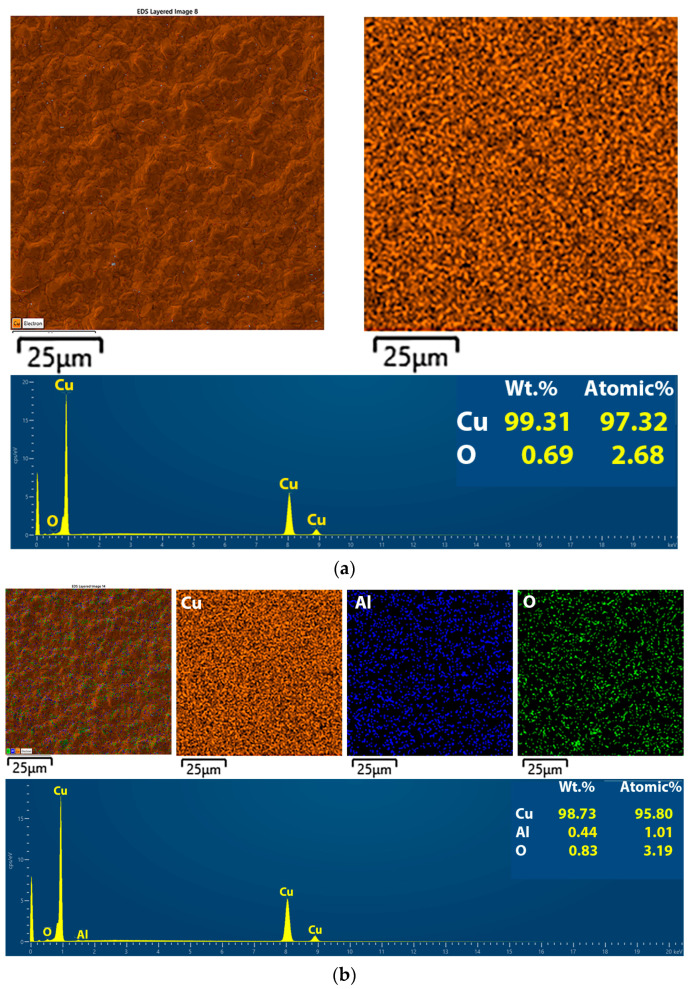
Element mapping of films: (**a**) alumina-free Cu film and (**b**) 5.0 wt.% of alumina particles. Film thickness was 22 μm.

Figure 7 shows the mapping analysis of alumina-free Cu, (Figure 7a) and Cu-Al_2_O_3_ films obtained at the same deposition times with 5.0 wt.% amounts of the alumina particles (Figure 7b).

Clear copper with dominant spectrum peaks was obtained from ABSEs. The atomic percentage of copper was 97.32%. The native oxide was present at 2.68% (Figure 7a). The surface area of the Cu-Al_2_O_3_ deposits shows the presence of elements from the alumina particles such as Al (0.32%) and O (1.35%) besides copper (98.32%) (Figure 7b). This suggests that the alumina particles were integrated or embedded in the copper film. To explain their integration into the deposit, the co-deposition of tiny inert particles floating in an electrolyte has generally been explained by the trapping process [31,32,33]. The EDS analysis of the films (thickness is 22 μm) with 1.0 wt.% and 3 wt.% alumina particles is shown in Figure S2a,b in the Supplementary Materials, respectively.

The EDS results of the elemental compositions for Cu-Al_2_O_3_ films co-electrodeposited with varying concentrations of alumina powder in the ABSE for constant film thickness (22 μm) are given in atomic percentages in Table 3.

The amount of particles incorporated in the MMC films was determined by the wet or counting method [13], but the EDS method was suitable for evaluating the elemental amount on the film surface. As the concentration of alumina in the electrolyte increases, an increase in the concentration of Al on the surface of the Cu film is observed [30]. The oxygen content also increases, but there is also the possibility of a lower affinity of copper to form the native oxide of copper films with the incorporation of alumina particles.

**Figure 8:**
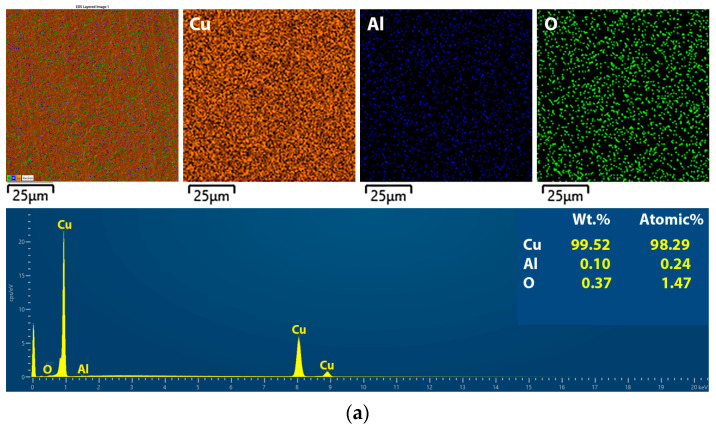

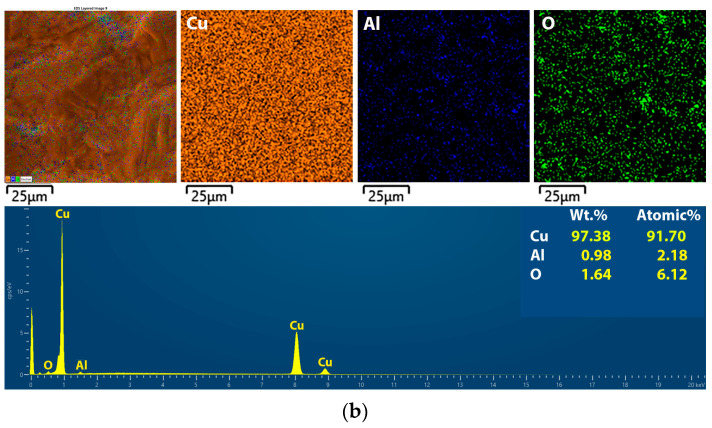
Element mapping of the Cu-Al_2_O_3_ films co-electrodeposited with a constant concentration of alumina powder (1.0 wt.%) in electrolytes with film thickness variation: (**a**) 2 μm and (**b**) 52 μm alumina powder.

Figure 8 shows the elemental mapping analysis of the Cu-Al_2_O_3_ films co-electrodeposited with a constant concentration of alumina powder (1.0 wt.%) in the electrolyte for minimum and maximum values of film thickness. The mapping and spectra are shown for a thickness of 2 μm Cu-Al_2_O_3_ (Figure 8a) and 52 μm Cu-Al_2_O_3_ (Figure 8b). The content of each element is given in atomic percent in Table 4.

From the values shown in Table 4, it can be concluded that as the thickness of the film increases, the percentage of alumina in the film increases, reducing the matrix element (copper). The nano-alumina powder has a strong tendency to aggregate in the electrolyte due to its extremely high surface energy and small primary size, resulting in nanoparticle aggregates that are easily embedded in the composites, despite the continuous stirring of the electrolyte before and during the deposition process [34]. This effect was enhanced and intensified by increasing the current density and particle concentration in the electrolyte [30]. The variations in the content of nano-alumina particles in the electrolyte also had a considerable effect on the thickness growth [35].

#### 2.2.3. Roughness Analyses of Free Cu and Cu-Al_2_O_3_ MMC Films—AFM Analyses

**Figure 9:**
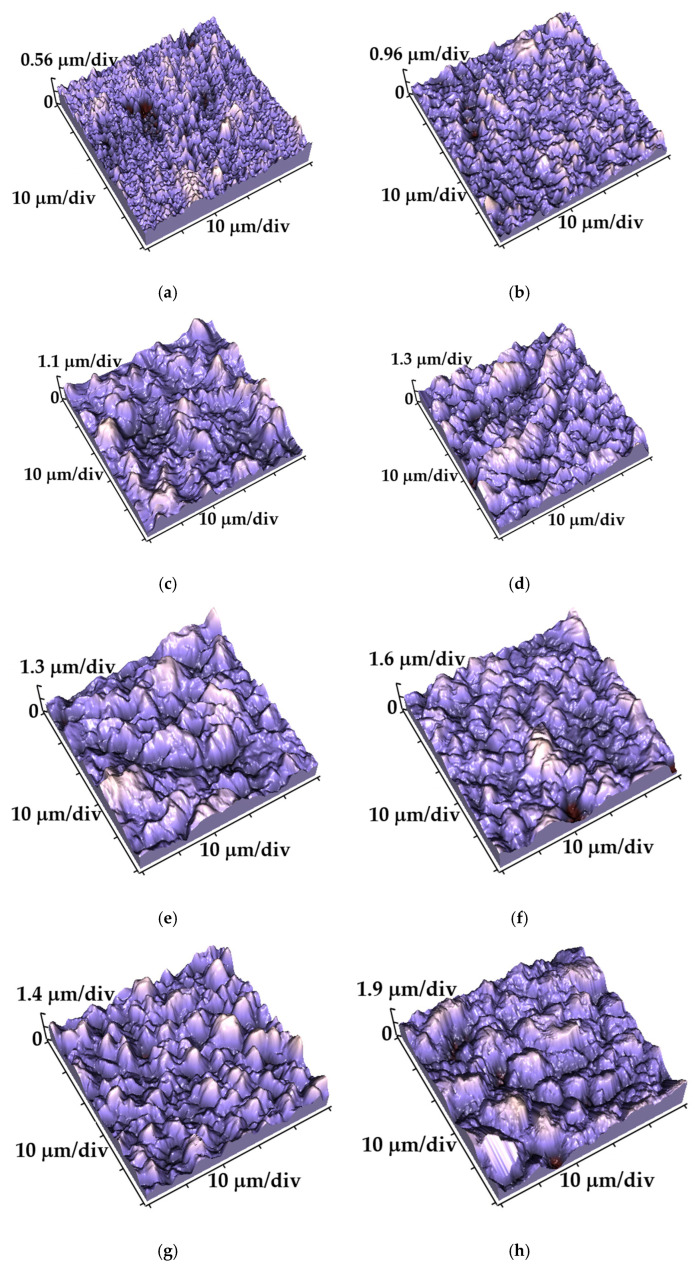
The 3D AFM images of all deposition Cu and Cu-Al_2_O_3_ films: (**a**) 2 μm Cu; (**b**) 2 μm Cu-Al_2_O_3_—1 wt.%; (**c**) 22 μm Cu; (**d**) 22 μm Cu-Al_2_O_3_—1 wt.%; (**e**) 52 μm Cu; (**f**) 52 μm Cu-Al_2_O_3_—1 wt.%; (**g**) 2 μm Cu-Al_2_O_3_—3%; and (**h**) 22 μm Cu-Al_2_O_3_—5 wt.%. The scan size was 50 × 50 μm^2^.

Figure 9 represents the 3D (three-dimensional) AFM images for the following films: the alumina-free Cu films with three different thicknesses (2, 22, and 52 μm) (Figure 9a,c,e) and the Cu-Al_2_O_3_ films with a variation of the same thickness: 2 μm (Figure 9b), 22 μm (Figure 9d), and 52 μm (Figure 9f). The variation in alumina concentration for Cu-Al_2_O_3_ films is shown in Figure 9g (for 3.0 wt.%) and in Figure 9h (for 5.0 wt.%). A light shade of purple corresponds to a maximum of grain peak, while a dark shade represent the base between grains.

The surface arithmetic means of the absolute roughness (*S*_a_) values, and the root mean square roughness parameter (*S*_q_) obtained using AFM software (SPMLab NT Ver. 6.0.2.) for the Cu films produced without/with alumina nanoparticles are given in Table 5.

The increase in roughness of the Cu and Cu-Al_2_O_3_ films is a result of several parameters: the roughening of the brass surface with chemical etching, the cathode surface with increasing electrodeposition time, the incorporation of alumina nanoparticles in the films, and the change in the incorporation process with changing the concentration of alumina particles in the suspension [36,37]. The maximum roughness (1066 nm) has sample co-electrodeposited from electrolyte ABSE-Al-5% for maximum thickness. The minimum roughness (96.5 nm) is for the sample without alumina with the 2 μm thick Cu film. The general conclusion is that rougher films are obtained with increasing deposition time and alumina concentration in the electrolyte. When comparing films of the same thickness with and without alumina particles, all composite films were found to be rougher than the pure Cu films, which is not always expected with nanoscale reinforcements.

#### 2.2.4. Microhardness Analyses of Free Cu and Cu-Al_2_O_3_ MMC Films—Vickers Indentation Method

**Figure 10:**
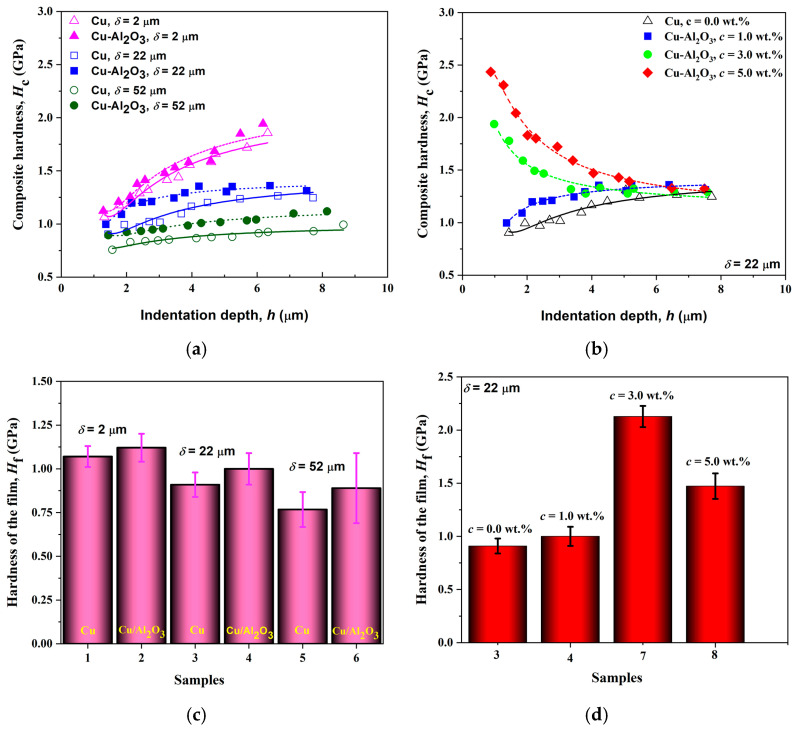
The microhardness analysis of Cu (normal line) and Cu-Al_2_O_3_ (dash line) MMC films applied to the C-G model: (**a**) with variations in the thickness of the films, (**b**) variations in the concentration of alumina particles in the suspension, and histograms with calculated values of an absolute hardness of the films (**c**) with different thicknesses, and (**d**) with 22 µm thickness and different concentrations of alumina particles.

Figure 10a, b present composite hardness data vs. indentation depth, *h*, together with fitting curves for all films (without/with alumina) and for three thicknesses (2, 22, and 52 μm), Figure 10a, and for all concentrations of alumina particles in the suspension, Figure 10b. The theoretical part related to the understanding of the composite hardness model is given in the Supplementary Materials. Figure 10c,d show histograms of the calculated and absolute microhardness, corrected for the influence of the brass substrate, for both cases of thickness and concentration variations. The phenomenological differences between the term’s microhardness, composite hardness, absolute hardness of the film, and absolute hardness of the substrate are defined in the Supplementary Materials.

For this purpose, the Chen–Gao composite hardness model was selected and applied [38]. The maximum hardness has the thinnest film reinforced with alumina nanoparticles. As the film thickness increases, the microhardness decreases. This can be explained by the transition from thin films to bulk material, which leads to an increase in the porosity and roughness of the film for free copper films. On the other hand, increasing the amount of alumina particles in the composite films seems to decrease film porosity [35]. However, there is also an increase in the standard error of measurement with increasing thickness, which can be explained by the appearance of agglomerates and the non-uniform distribution of the alumina particles on the surface of the film [39]. All Cu films and Cu-Al_2_O_3_ composite films with 1.0 wt.% alumina particles belong to the “soft film on hard substrate” composite system type, and all curves have a rising character in Figure 10a. The situation changes when it comes to Cu-Al_2_O_3_ composite films synthesized with 3.0 and 5.0 wt.% of alumina particles in the electrolyte. There is a change in the type of composite system (red and green lines in Figure 10b). The addition of Al_2_O_3_ nanoparticles during co-electrodeposition synergistically increased the hardness by about 4.73% for 2 µm thick films, 9.96% for 22 µm thick films, and 15.9% for 52 µm thick films, compared to pure Cu and Cu-Al_2_O_3_ films (Figure 10c).

The increasing Al_2_O_3_ nanoparticle concentrations during co-electrodeposition increased the hardness by about 9.96% for 1 wt.%, 134.1% for 3.0 wt.%, and 61.9% for 5.0 wt.%, compared to pure Cu films (Figure 10d). The consequences of this change can be explained through the change in film adhesion and the dispersion hardening effect. Thus, the higher density of grain boundaries in the nanocrystalline materials, which slows down the dislocation motion, can be linked to the hardening. The relatively soft copper matrix’s plastic flow will be inhibited by the nanoparticles. However, an increase in hardness does not always correlate with grain size reduction or an increase in the content of incorporated particles within the film. Several additional factors play crucial roles, including surface roughness, grain shape, grain distribution, film thickness, porosity, the presence or absence of embedded agglomerates of particles, and film–substrate interfacial adhesion. Alumina, a brittle ceramic particle, especially in nanoscale form, tends to agglomerate within the Cu matrix due to its large specific surface area and the influence of van der Waals forces [40]. The second issue is the poor wettability between the alumina reinforcement phase and the copper matrix, leading to weak interfacial bonding. However, the presence of oxides on the surface of the composite films, derived from both alumina particles and natural copper oxide, can effectively enhance hardness while preserving plasticity.

A comparative study of the change in the microhardness of the copper matrix with the incorporation of alumina particles is shown in Table 6, where data are compared to other similar studies. For pure copper, the film had a microhardness of 80 HV (0.78 GPa), while composite films had a microhardness of 310 HV (3.04 GPa) [41]. According to published research, the hardness of Cu films from copper sulphate baths ranges from 50 to 105 HV (0.49–1.47 GPa) [42], depending on the electrolyte composition and direct current (DC) plating parameters. Values of nanohardness for pure Cu films, compared to DC plating and PC plating, have produced copper deposits that are five times larger [43]. The correlation between the hardness of metal films and composite films and their microstructure in addition to the amount and dispersion of the reinforcing stage were given in our previous publications [16,44].

#### 2.2.5. Adhesion Analyses of Free Cu and Cu-Al_2_O_3_ MMC Films—Vickers Indentation Method

The adhesion of thin films is a very important feature that often determines the physical existence of a film on a substrate. Films that do not have good adhesion are delaminated from the substrate, and such films have no use value when it comes to microelectronic applications and electrical packaging. Testing the adhesion properties of the film can be achieved through the Vickers microindentation test, with theoretical approximations and the application of mathematical models. The basic theoretical equations of the model and the meanings of the variables are given in the Supplementary Materials.

**Figure 11:**
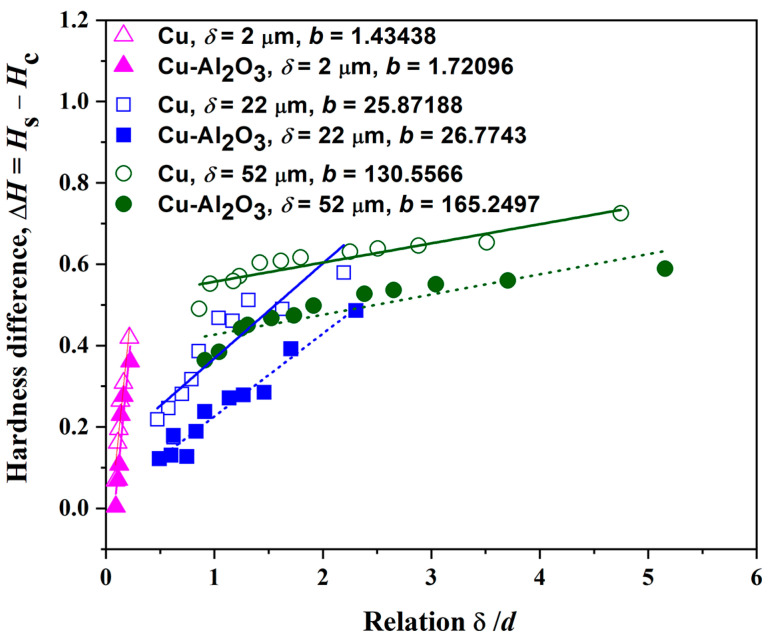
Adhesion properties of the films without/with reinforced alumina. Values of the adhesion parameter, called “critical reduced depth” for Cu (normal line) and Cu-Al_2_O_3_ (dashed line) are also given.

Figure 11 shows the dependency of hardness difference, Δ*H*, on a ratio, *δ*/*d*, for the Cu films made from the electrolyte ABSE, and for the composite films Cu/Al_2_O_3_ made from electrolyte ABSE-Al-1%. A comparison of an adhesion parameter, *b*, for all three thicknesses, was also given. Table 7 provides the calculated values for the adhesion parameter, *b*, which was determined based on the slope of the linear dependencies depicted in Figure 11. The greater the adhesion parameter *b* value, the more the films adhere to the substrate [18].

Table 7 illustrates that Cu-Al_2_O_3_ films formed from electrolyte ABSE-Al-1% demonstrated superior adherence compared to those obtained without alumina particles (electrolyte ABSE). Concurrently, the Cu films and Cu-Al_2_O_3_ films generated at a shorter deposition time (thinner films) from both electrolytes had the poorest adherence with the cathode surface. The value of composite microhardness data is influenced by the adhesion of the film to the cathode: stronger adhesion corresponds to higher hardness of the composite [46]. For harder films, the deformation zone at the interface of film/substrate is more extended, and critical reduced depth has a higher value for the “soft film on a hard substrate” type. This rule applies to the minimum concentration of alumina (1.0 wt.%). However, with Cu-Al_2_O_3_ composite films co-electrodeposited from electrolytes with 3.0 and 5.0 wt.% alumina particles (electrolyte ABSE-Al-3% and ABSE-Al-5%), there is a change in the type of composite system. By incorporating alumina particles, the composite film becomes harder than the brass substrate. An obvious decrease in the adhesion parameter was noted with an increase in the concentration of alumina in the electrolyte. Based on Lawn’s law and the “spherical field stress model” [47], the contribution of the elastic component for both materials (films and substrate), expressed through Jung’s modulus of elasticity *E*, as well as the contribution of the plastic component for both materials, expressed through hardness *H*, must be considered. Ceramic particles generally reduce the elasticity of metallic films [48], so the plastic component increases. However, ceramic particles with a high specific elastic modulus can effectively increase Young’s modulus of the Cu composites [49]. The radius of the deformation zone under the indenter increases, so, critical reduced depth (*b*) decreases, too. Precisely because of this, there is a decrease in adhesion at a higher alumina concentration in the film, although the hardness of the film shows a higher value than the copper matrix.

#### 2.2.6. Wettability Analyses of Free Cu and MMC Cu-Al_2_O_3_ Films—Sessile Drop Method

**Figure 12:**
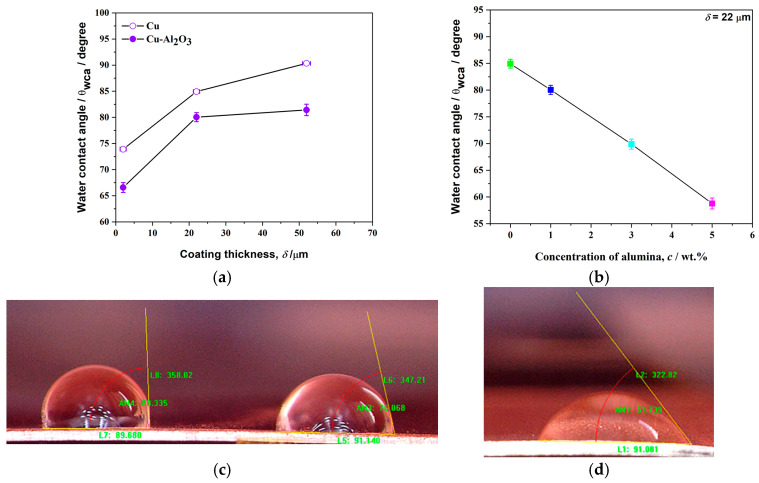
The values of static contact angles measured on the surface of Cu and Cu-Al_2_O_3_ composite films: (**a**) with varying thicknesses and 1 wt.% of alumina particles, (**b**) with variations in the concentration of alumina particles in ABSEs, (**c**) with the appearance of a water droplet on a particle-free copper film, and (**d**) with 5.0 wt.% of alumina particles.

It is known that surface roughness directly affects the surface’s wettability [19,50]. Measuring the static contact angles using different liquids is useful to determine the films’ and composite films’ wettability and affinity to polar and non-polar components. A sessile drop technique has been used to measure the water contact angle. The water contact angle (*θ*_WCA_) of the pure Cu films and Cu-Al_2_O_3_ films at various thicknesses of the films were measured, and the results are shown in Figure 12a.

The relationship between the concentration of alumina particles in the electrolyte and the impact on the water contact angle value of Cu and Cu-Al_2_O_3_ composite films is given in Figure 12b. Figure 12c,d show the method of measuring water contact angles on the surface of copper and MMC Cu-Al_2_O_3_ films with the maximum content of alumina particles.

The copper films without alumina particles electrodeposited on the brass substrate were hydrophilic and had a water contact angle value of less than 90° (*θ*_WCA_ = 84.94 ± 0.83). A more hydrophilic characteristic is exhibited by Cu-Al_2_O_3_ composite films that are produced by the co-deposition method. On the surface area of MMC Cu-Al_2_O_3_ films, the measured water contact angles ranged from 66.57 ± 0.94° to 81.42 ± 1.10° (Figure 12a). As demonstrated in our instance, rougher films, i.e., thicker films, are generally more hydrophobic than smooth and thick films [50]. Therefore, a change in the wettability of films results from an increase in film roughness with thickness variation. The lower contact angle of the Cu-Al_2_O_3_ films in comparison to the free Cu films may be due to the particle’s incorporation on the surface of films and their relatively polar nature. On the other hand, it is known that ceramic particles increase the porosity of the composite [51], so this is an additional reason for better absorption of water. By altering the quantity of alumina particles in the electrolyte, the Cu-Al_2_O_3_ films’ water contact angle changed (see Figure 12b). Although an increase in the roughness of composite films with the incorporation of alumina particles was recorded, here, in addition to the roughness, a more dominant factor on wetting is observed, namely the presence of polar alumina particles on the surface. With the increase in the concentration of alumina in the electrolyte to 5.0 wt.%, the value of the water contact angle drops from 84.94 ± 0.83° to 58.78 ± 1.03°. Numerous polar sites from unsaturated oxygen and aluminum atoms, which function as potent Lewis’s acid and base sites, respectively, are present in alumina [52]. These surface aluminum atoms quickly create hydrophilic hydration structures by hydrogen bonding with interfacial water molecules [53]. Also, surface polarity is significantly increased by the substantial presence of unsaturated ions on the surface [54,55]. Hence, increased surface wettability is greatly influenced by the oxidation processes of copper films and the presence of oxide ceramic particles incorporated in the Cu matrix. Compared to our previous research on the values of the water contact angle, for the MMC Cu film reinforced with micro-sized pigment particles based on strontium aluminate, the water contact angles are very close to each other, in the range of 106.0–110.8°, but about 60% larger than for the pure Cu coating [16]. In this case, we had a transition from a hydrophilic free-copper film to a hydrophobic one. Identical wetting behaviour is shown for reinforced copper films with Fe/Al LDO (ferrite–aluminium-layered double oxide) nanoparticles [44]. The wettability of the Cu films changed from hydrophilic (for pure Cu) to hydrophobic (for Cu-Fe/Al LDO), and this change was dominated by an increase in the roughness parameter of the films.

In this case, the surface contact angles depend on the microstructure, roughness, presence of particles, type and size of particles, and film thickness. It is found that surface wettability increases as the roughness, thickness, and alumina concentration increases. As the parameters increase, the nature of the films becomes more hydrophilic. The dominant factor in this behaviour is the nature of the synthesized alumina particles and their presence on the top surface of the copper films [56].

## 3. Conclusions

Alumina particles were obtained from the sol–gel process from inorganic precursors. The particles had a corundum structure obtained at 1000 °C and a submicron size. Individual particles were small but tended to make agglomerates that could influence the process when longer deposition times and thicker films were made. Alumina particles were incorporated in copper films, and for thinner films, they increased the hardness of films, and the hardness for thicker films was decreased. From the point of view of hardness improvement, the optimal value for films is 22 µm. The incorporation of alumina also depends on the concentration of powder particles in the electrolyte, and the concentration of alumina particles in the film is dependent on this parameter. Adhesion of the film on the substrate depends both on the film thickness and alumina content. For the same film thickness, a pure copper film has a lower adhesion value compared to the composite film. The best adhesion for MMC films was obtained for the optimal alumina concentration in ABSEs, which is 1.0 wt.%. Roughness increases both with the film thickness and the content of alumina particles incorporated in the film. The grain size of copper films has the same trend as the roughness, showing an increasing character with increasing film thickness and with the incorporation of alumina particles. The wettability of the material is better when alumina particles are incorporated into the film, and this can be useful for some potential applications of composite films on brass substrates.

This sort of composite film coating, with improved hardness and wettability, is very suitable for microfluidic devices used for the trybocatalytic removal of pollutants in corrosive media. The industrial application of this type of composite film could also be useful in the construction of microheaters, which are used for the production of reactors, where it is necessary for the material to be resistant to corrosion and have improved resistance to the environment and at the same time assure the improved heat transfer conditions due to improved surface characteristics.

## 4. Materials and Methods

### 4.1. Materials and Method—Synthesis of Al_2_O_3_ Nanoparticles

The Clariant firm provided aluminum hydroxide chloride (Locron L; Al_2_Cl(OH)_5_ × 2.5 H_2_O) in its crystallized form for purchase as an alumina precursor. Aluminum oxide Al_2_O_3_ nanoparticles were synthesized using the sol–gel technique. Al_2_Cl(OH)_5_ × 2.5 H_2_O was dissolved in demineralized water with a magnetic stirrer. They formed a sol that was transformed into a gel. The gel was heat treated at 1000 °C to obtain a suitable crystal structure for matrix reinforcement and to obtain fine small particles.

### 4.2. Materials and Method—Synthesis of Cu Films

Cold-rolled brass foil (ASTM B36 (70% Zn and 30% Cu)), K&S Engineering, Chicago, IL, USA), 250 µm thick, was used as a cathode, and copper foil, 1 mm thick (Alfa Aesar ThermoFisher GmbH Erlenbachweg, Kandel, Germany), was used as an anode.

The solution of a mixture of three acids (HNO_3_:H_3_PO_4_:CH_3_COOH = 4:11:5 vol.% at 10 s) was used for chemical polishing after mechanical polishing with SiC paper (#2000) for the preparation cathode. A solution of nitric acid (Merck KGaA, Darmstadt, Germany) in HNO_3_:H_2_O = 1:1 vol.% was used for the preparation anode. Copper (II)-sulphate pentahydrate, CuSO_4_·5H_2_O, (Merck KGaA, Darmstadt, Germany), and sulphuric acid (98%) of p.a. quality were ordered from Zorka, Šabac, Serbia. The high-purity water, 18 MΩ·cm (Milipore, Burlington, MA, USA), was used for the preparation of an acidic basic sulphate electrolyte (ABSE). The cathodes with a total area of 5.0 × 1.0 cm^2^, and an effective deposition surface area of 2.0 × 1.0 cm^2^, were put in the middle of the electrolytic cell. The magnetic stirrer was used for the mixing of the electrolyte at 200 rpm. An ABSE composition of 240 g/L CuSO_4_⋅5 H_2_O and 60 g/L H_2_SO_4_ was put in the 100 mL Pyrex glass. The pH values and temperature were 0.33 and 20 ± 0.5 °C, respectively. The constant current regime (galvanostatically) was turned off on the Keithley 2200 DC Power Supply Programmable device (Tektronix UK Ltd., Bracknell, UK). Based on Faraday’s law, the deposition times were calculated to obtain the projected film’s thicknesses of 2, 22, and 52 μm.

### 4.3. Materials and Method—Synthesis of Cu-Al_2_O_3_ Composite Films

A co-electrodeposition method was performed by the addition of Al_2_O_3_ nanoparticles (synthesized and described in Section 4.1) into the ABSE, and these electrolytes were noted as ABSE-Al electrolytes. Three concentrations of alumina particles in ABSEs were used (1.0, 3.0, and 5.0 wt.%) and electrolytes were marked as ABSE-Al-1%, ABSE-Al-3%, and ABSE-Al-5%. These electrolytes were stirred for 120 min prior to the co-electrodeposition process (CED). The current density value was identical for both conditions, without and with added nanoparticles. After achieving better dispersion and wetting of particles in the electrolyte, i.e., prevention of agglomerates, the mixing intensity was reduced to 200 rpm.

### 4.4. Characterization Methods

All experimental and measurement set data are given in the Supplementary Materials.

## Figures and Tables

**Table 1:** Fractional coordinates for O and Al obtained according to XRD analyses of alumina particles produced using sol–gel method.

Fractional Coordinates for O and Al	*x*	*y*	*z*
O	0.68998 (38)	0.00000 (0)	0.25000 (0)
Al	0.00000 (0)	0.00000 (0)	0.35273 (10)

**Table 2:** The thickness and average grain size of Cu and Cu-Al_2_O_3_ films obtained from FE-SEM images.

Sample No.	Electrolyte	Experimental Thickness of Films, *δ* (µm)	Grain Size, D_mean_ (µm)
1.	ABSE	2	0.752
2.	ABSE-Al-1%	2	0.920
3.	ABSE	22	1.123
4.	ABSE-Al-1%	22	1.961
5.	ABSE	52	2.534
6.	ABSE-Al-1%	52	4.094
7.	ABSE-Al-3%	22	4.159
8.	ABSE-Al-5%	22	4.466

**Table 3:** The EDS results (atomic %) for the Cu-Al_2_O_3_ films co-electrodeposited with constant thickness (22 μm) and varying concentrations of alumina powder in the electrolyte.

Sample No.	Electrolyte	*c* (wt.%)	Elements		
			Al	O	Cu
2.	ABSE	0	0	2.68	97.32
4.	ABSE-Al-1%	1.0	0.35	0.68	98.97
7.	ABSE-Al-3%	3.0	0.54	1.52	97.95
8.	ABSE-Al-5%	5.0	1.01	3.19	95.80

**Table 4:** The EDS results for the Cu-Al_2_O_3_ films co-electrodeposited with a constant concentration of alumina powder (1.0 wt.%) and thickness variation.

Sample No.	Electrolyte	Experimental Thickness ofFilms, *δ* (µm)	Elements		
			Al	O	Cu
2.	ABSE-Al-1%	2	0.24	1.47	98.29
4.	ABSE-Al-1%	22	0.35	0.68	98.97
7.	ABSE-Al-1%	52	2.18	6.12	91.70

**Table 5:** The values of roughness parameter *S*_a_ obtained by application of the AFM software from a 50 × 50 µm^2^ scan area for the free-Cu and Cu-Al_2_O_3_ films with variations in the thicknesses of the films and concentrations of alumina powder in electrolytes.

*δ* (µm)	2	2	22	22	52	52	22	22
Electrolyte	ABSE	ABSE-Al-1%	ABSE	ABSE-Al-1%	ABSE	ABSE-Al-1%	ABSE-Al-3%	ABSE-Al-5%
*S*_a_ (nm)	96.5	312.2	153.8	354.7	366.6	584.7	420.7	849.0
*S*_q_ (nm)	126.7	387.1	195.8	436.7	452.4	695.8	519.6	1066.0

**Table 6:** Microhardness data of Cu and MMC Cu-Al_2_O_3_ films: ED—electrodeposition; *c—*concentration; CED—co-electrodeposition; DC—direct current mode; PC—pulse current mode; HV—Vickers hardness.

System Film/Substrate	*c*	Alumina Type	Method of Synthesis	Parameter of Synthesis	Method of Characterization	Hardness	Ref.
Cu/Cu	0	/	ED/DC	11 A/dm^2^, 20 min.	HV	81	[30]
Cu/Al_2_O_3_/Cu	30 g/L	Commercial/287 nm	CED/DC	11 A/dm^2^, 20 min.	HV	247	[30]
Cu/Cu	0	/	ED/DC	10 mA/cm^2^, 10 min.	HV	85	[35]
Cu/Al_2_O_3_/Cu	15.2 wt.%	Commercial/50 nm	CED/PC	10 mA/cm^2^	HV	149	[35]
Cu/Al_2_O_3_/Cu	17.2 wt.%	Commercial/50 nm	CED/PC	10 mA/cm^2^	HV	150	[35]
Cu/Al_2_O_3_/Cu	17.8 wt.%	Commercial/50 nm	CED/PC	10 mA/cm^2^	HV	156	[35]
Cu/Si	0	/	ED/DC	200 A/m^2^	Nanoindentation/GPa	1.29 ± 0.18	[39]
Cu/Al_2_O_3_/Si	5 g/l	Commercial/50 nm	CED/DC	200 A/m^2^	Nanoindentation/GPa	1.71 ± 0.14	[39]
Cu/Cu	0	/	ED/DC	10 mA/cm^2^	HV	100.4	[41]
Cu/Cu	0	/	ED/DC	75 mA/cm^2^	HV	138.2	[41]
Cu/Al_2_O_3_/Cu	8.2 wt.%	50 nm γ-Al_2_O_3_	CED/DC	10 mA/cm^2^	HV	182.9	[41]
Cu/Al_2_O_3_/Cu	3.2 wt.%	50 nm γ-Al_2_O_3_	CED/DC	75 mA/cm^2^	HV	241.7	[41]
Cu/Cu	0	/	ED/DC	4 ± 0.1 A/dm^2^	Rockwell	≈65	[45]
Cu/Al_2_O_3_/Cu	50 g/l	Commercial/50 nm	CED/DC	4 ± 0.1 A/dm^2^	Rockwell	≈50	[45]
Cu/Al_2_O_3_	1.0 wt.% 3.0 wt.% 5.0 wt.%	Synthesis from sol–gel	CED/DC	50 mA·cm^−2^	HV	101.9 217.0 150.1	This work

**Table 7:** Fitting results of adhesion parameter *b* for the alumina-free Cu film and those produced with different film thicknesses and concentrations in the electrolyte.

Concentration of Alumina, *c*/wt.%	Thickness, *δ*/μm	Slope (*k*)	Intercept (*n*)	*b*	*R* ^2^
0	2	2.74958 ± 0.47254	−0.2128 ± 0.06956	1.4344	0.86793
1.0	2	2.6125 ± 0.29533	−0.12388 ± 0.0425	1.7210	0.93920
0	22	0.20341 ± 0.01504	0.02246 ± 0.0183	25.872	0.94787
1.0	22	0.23341 ± 0.0388	0.13497 ± 0.0444	26.774	0.77871
0	52	0.0495 ± 0.00782	0.37686 ± 0.0197	130.56	0.78104
1.0	52	0.04716 ± 0.00644	0.50972 ± 0.0152	165.25	0.82692
3.0	22	−0.48371 ± 0.0421	0.43142 ± 0.0532	15.330	0.98821
5.0	22	−0.21415 ± 0.0537	0.38714 ± 0.0572	4.6713	0.84983

## Data Availability

The original contributions presented in the study are included in the article/supplementary material, further inquiries can be directed to the corresponding authors.

## Supplementary Materials

The following supporting information can be downloaded at https://www.mdpi.com/article/10.3390/gels10100648/s1: Figure S1: Surface morphology and histogram analyses of electrodeposited MMC Cu-Al_2_O_3_ films co-electrodeposited on the brass substrate with a constant thickness of the films (22 µm) and with different concentrations of alumina particles in an ABSE: (a) 3.0 wt.% and (b) 5.0 wt.%. The magnification was × 10,000 for all pictures. The co-electrodeposition mode was DC with a current density of 50 mA·cm^−2^; Figure S2: Element mapping of the MMC Cu-Al_2_O_3_ films co-electrodeposited on the brass substrate with a constant thickness of the films (22 µm) and various concentrations of alumina particles in an ABSE: (a) 1.0 wt.% and (b) 3.0 wt.%. The film thickness was 22 µm. Reference [38] is in the Supplementary Materials as number [12].

## References

[1] Kaunisto K., Lagerbom J., Honkanen M., Varis T., Lambai A., Mohanty G., Levänen E., Kivikytö-Reponen P., Frankberg E. (2023). Evolution of Alumina Phase Structure in Thermal Plasma Processing. Ceram. Int..

[2] Abyzov A.M. (2019). Aluminum Oxide and Alumina Ceramics (Review). Part 1. Properties of Al2O3 and Commercial Production of Dispersed Al2O3. Refract. Ind. Ceram..

[3] Boumaza A., Favaro L., Lédion J., Sattonnay G., Brubach J.B., Berthet P., Huntz A.M., Roy P., Tétot R. (2009). Transition Alumina Phases Induced by Heat Treatment of Boehmite: An X-Ray Diffraction and Infrared Spectroscopy Study. J. Solid State Chem..

[4] Levin I., Brandon D. (1998). Metastable Alumina Polymorphs: Crystal Structures and Transition Sequences. J. Am. Ceram. Soc..

[5] Drah A., Tomić N.Z., Kovačević T., Djokić V., Tomić M., Heinemann R.J., Marinković A. (2020). Structurally and Surface-Modified Alumina Particles as a Reinforcement in Polyester-Based Composites with an Improved Toughness. Mech. Compos. Mater..

[6] Ashor A.A., Vuksanović M.M., Tomić N.Z., Petrović M., Dojčinović M., Husović T.V., Radojević V., Heinemann R.J. (2019). Optimization of Modifier Deposition on the Alumina Surface to Enhance Mechanical Properties and Cavitation Resistance. Polym. Bull..

[7] Lazouzi G.A., Vuksanović M.M., Tomić N., Petrović M., Spasojević P., Radojević V., Jančić Heinemann R. (2019). Dimethyl Itaconate Modified PMMA—Alumina Fillers Composites with Improved Mechanical Properties. Polym. Compos..

[8] Santos J.S., Márquez V., Buijnsters J.G., Praserthdam S., Praserthdam P. (2023). Antimicrobial Properties Dependence on the Composition and Architecture of Copper-Alumina Coatings Prepared by Plasma Electrolytic Oxidation (PEO). Appl. Surf. Sci..

[9] Mitra D., Kang E.-T., Neoh K.G. (2020). Antimicrobial Copper-Based Materials and Coatings: Potential Multifaceted Biomedical Applications. ACS Appl. Mater. Interfaces.

[10] Kumykov V.K., Sergeev I.N., Sozaev V.A., Gedgagova M.V. (2017). Surface Tension of Copper in Solid Phase. Bull. Russ. Acad. Sci. Phys..

[11] Podlaha E.J., Landolt D. (1997). Pulse-Reverse Plating of Nanocomposite Thin Films. J. Electrochem. Soc..

[12] Thiemig D., Lange R., Bund A. (2007). Influence of Pulse Plating Parameters on the Electrocodeposition of Matrix Metal Nanocomposites. Electrochim. Acta.

[13] Stankovic V.D., Gojo M. (1996). Electrodeposited Composite Coatings of Copper with Inert, Semiconductive and Conductive Particles. Surf. Coatings Technol..

[14] Wang C., Bai L., Xu H., Qin S., Li Y., Zhang G. (2024). A Review of High-Temperature Aerogels: Composition, Mechanisms, and Properties. Gels.

[15] Dascalu I., Hornoiu C., Calderon Moreno J.M., Osiceanu P., Somacescu S. (2023). Layered Sol–Gel Deposition of a Sn, Ti, Zn, and Pr Mixed Oxide Thin Film with Electrical Properties for Gas Sensing. Gels.

[16] Mladenović I.O., Vuksanović M.M., Dimitrijević S.P., Vasilić R., Radojević V.J., Vasiljević-Radović D.G., Nikolić N.D. (2023). Mechanical Properties of Electrolytically Produced Copper Coatings Reinforced with Pigment Particles. Metals.

[17] Algellai A.A., Tomić N., Vuksanović M.M., Dojčinović M., Volkov-Husović T., Radojević V., Heinemann R.J. (2018). Adhesion Testing of Composites Based on Bis-GMA/TEGDMA Monomers Reinforced with Alumina Based Fillers on Brass Substrate. Compos. Part B Eng..

[18] Mladenović I.O., Bošković M.V., Vuksanović M.M., Nikolić N.D., Lamovec J.S., Vasiljević-Radović D.G., Radojević V.J. (2022). Structural, Mechanical and Electrical Characteristics of Copper Coatings Obtained by Various Electrodeposition Processes. Electronics.

[19] Mogra A., Pandey P.K., Gupta K.K., Shivhare S., Bagal V. (2022). Development and Characterization of Cu-Al2O3 Nanocomposite Coating Using Electrodeposition Process on Copper Substrate. J. Inst. Eng. Ser. C.

[20] Souza H.J.D., D’Souza N., Ashith V.K., Nagappa Moger S., D’Silva E.D. (2023). Effect of Deposition Time on Copper Incorporation of ZnS Thin Films by Low-Cost Technique. Mater. Sci. Eng. B.

[21] Mladenović I.O., Nikolić N.D. (2023). Influence of Parameters and Regimes of the Electrodeposition on Hardness of Copper Coatings. Metals.

[22] Alazreg A., Vuksanović M.M., Egelja A., Mladenović I.O., Radovanović Ž, Petrović M., Marinković A., Jančić Heinemann R. (2023). Mechanical properties of acrylate matrix reinforced with manganese-aluminum layered double hydroxide (MnAl-LDH). Polym. Compos..

[23] Gafur M.A., Al-Amin M., Sarker M.S.R., Alam M.Z. (2021). Structural and Mechanical Properties of Alumina-Zirconia (ZTA) Composites with Unstabilized Zirconia Modulation. Mater. Sci. Appl..

[24] Petrík J., Blaško P., Markulík Š., Šolc M., Palfy P. (2022). The Indentation Size Effect (ISE) of Metals. Crystals.

[25] Gong J., Wu J., Guan Z. (1999). Examination of the Indentation Size Effect in Low-Load Vickers Hardness Testing of Ceramics. J. Eur. Ceram. Soc..

[26] Li N., Liu L., Zhang M. (2009). The Role of Friction to the Indentation Size Effect in Amorphous and Crystallized Pd-Based Alloy. J. Mater. Sci..

[27] Li H., Bradt R.C. (1993). The Microhardness Indentation Load/Size Effect in Rutile and Cassiterite Single Crystals. J. Mater. Sci..

[28] Chuah H.G., Ripin Z.M. (2013). Quantifying the Surface Roughness Effect in Microindentation Using a Proportional Specimen Resistance Model. J. Mater. Sci..

[29] LaGraff J.R., Gewirth A.A. (1995). Nanometer-Scale Mechanism for the Constructive Modification of Cu Single Crystals and Alkanethiol Passivated Au(111) with an Atomic Force Microscope. J. Phys. Chem..

[30] Maharana H.S., Ashok A., Pal S., Basu A. (2016). Surface-Mechanical Properties of Electrodeposited Cu-Al2O3 Composite Coating and Effects of Processing Parameters. Metall. Mater. Trans. A.

[31] Guglielmi N. (1972). Kinetics of the Deposition of Inert Particles from Electrolytic Baths. J. Electrochem. Soc..

[32] Li Y.J., Zhang X.Z., Zhi C.C. (2021). Kinetics of Ni/Nano-SiO2 Codeposition on the Sintered NdFeB Surface. Strength Mater..

[33] Dordsheikh Torkamani A., Velashjerdi M., Abbas A., Bolourchi M., Maji P. (2021). Electrodeposition of Nickel Matrix Composite Coatings via Various Boride Particles: A Review. J. Compos. Compd..

[34] Kumar N., Kishore K., Yadav S., Sharma P. (2024). Characterisation of Ni-Al2O3 Composite Coatings at Different Al2O3 Concentrations. Mater. Today Proc..

[35] Allahkaram S.R., Golroh S., Mohammadalipour M. (2011). Properties of Al2O3 Nano-Particle Reinforced Copper Matrix Composite Coatings Prepared by Pulse and Direct Current Electroplating. Mater. Des..

[36] Lim J.D., Susan Y.S.Y., Daniel R.M., Leong K.C., Wong C.C. (2013). Surface Roughness Effect on Copper–Alumina Adhesion. Microelectron. Reliab..

[37] Yang J., Huang Y., Xu K. (2007). Effect of Substrate on Surface Morphology Evolution of Cu Thin Films Deposited by Magnetron Sputtering. Surf. Coatings Technol..

[38] Chen M., Gao J. (2000). The Adhesion of Copper Films Coated on Silicon and Glass Substrates. Mod. Phys. Lett. B.

[39] Kim M., Sun F., Lee J., Hyun Y.K., Lee D. (2010). Influence of Ultrasonication on the Mechanical Properties of Cu/Al2O3 Nanocomposite Thin Films during Electrocodeposition. Surf. Coatings Technol..

[40] Yan Y.-F., Kou S.-Q., Yang H.-Y., Shu S.-L., Qiu F., Jiang Q.-C., Zhang L.-C. (2023). Ceramic Particles Reinforced Copper Matrix Composites Manufactured by Advanced Powder Metallurgy: Preparation, Performance, and Mechanisms. Int. J. Extrem. Manuf..

[41] Thiemig D., Osborne S., Sweet W., Talbot J. (2008). Electroplating of Copper-Alumina Nanocomposite Films with an Impinging Jet Electrode. ECS Trans..

[42] Lamb V.A., Johnson C.E., Valentine D.R. (1970). Physical and Mechanical Properties of Electrodeposited Copper. J. Electrochem. Soc..

[43] Tao S., Li D.Y. (2006). Tribological, Mechanical and Electrochemical Properties of Nanocrystalline Copper Deposits Produced by Pulse Electrodeposition. Nanotechnology.

[44] Sasi Maoloud Mohamed S., Nikolić N.D., Vuksanović M.M., Vasilić R., Vasiljević-Radović D.G., Jančić Heinneman R.M., Marinković A.D., Mladenović I.O. (2024). Hardness and Wettability Characteristics of Electrolytically Produced Copper Composite Coatings Reinforced with Layered Double Oxide (Fe/Al LDO) Nanoparticles. Coatings.

[45] Tey E., Hashim M., Ismail I. (2016). Characterization of Cu-Al2O3 and Ni-Al2O3 Nanocomposites Electrodeposited on Copper Substrate. Mater. Sci. Forum.

[46] Magagnin L., Maboudian R., Carraro C. (2003). Adhesion Evaluation of Immersion Plating Copper Films on Silicon by Microindentation Measurements. Thin Solid Films.

[47] Lesage J., Chicot D. (1999). Models for Hardness and Adhesion of Coatings. Surf. Eng..

[48] Hsieh C.L., Tuan W.H. (2005). Elastic Properties of Ceramic–Metal Particulate Composites. Mater. Sci. Eng. A.

[49] Hashin Z., Shtrikman S. (1963). A Variational Approach to the Theory of the Elastic Behaviour of Multiphase Materials. J. Mech. Phys. Solids.

[50] Wang S., Feng L., Liu H., Sun T., Zhang X., Jiang L., Zhu D. (2005). Manipulation of Surface Wettability between Superhydrophobicity and Superhydrophilicity on Copper Films. ChemPhysChem.

[51] Samal P., Vundavilli P.R., Meher A., Mahapatra M.M. (2020). Recent Progress in Aluminum Metal Matrix Composites: A Review on Processing, Mechanical and Wear Properties. J. Manuf. Process..

[52] Cardoso J.T., Garcia-Girón A., Romano J.M., Huerta-Murillo D., Jagdheesh R., Walker M., Dimov S.S., Ocaña J.L. (2017). Influence of Ambient Conditions on the Evolution of Wettability Properties of an IR-, Ns-Laser Textured Aluminium Alloy. RSC Adv..

[53] Argyris D., Ashby P.D., Striolo A. (2011). Structure and Orientation of Interfacial Water Determine Atomic Force Microscopy Results: Insights from Molecular Dynamics Simulations. ACS Nano.

[54] Li W., Jin Y., Gu J., Zeng Z., Su X., Xu J., Guo B. (2024). Critical Surface Characteristics for Coating Adhesion and Friction Behavior of Aluminum Alloys after Laser Cleaning. J. Mater. Process. Technol..

[55] Tong W., Cui L., Qiu R., Yan C., Liu Y., Wang N., Xiong D. (2021). Laser Textured Dimple-Patterns to Govern the Surface Wettability of Superhydrophobic Aluminum Plates. J. Mater. Sci. Technol..

[56] Gun’ko V.M., Yurchenko G.R., Turov V.V., Goncharuk E.V., Zarko V.I., Zabuga A.G., Matkovsky A.K., Oranska O.I., Leboda R., Skubiszewska-Zięba J. (2010). Adsorption of Polar and Nonpolar Compounds onto Complex Nanooxides with Silica, Alumina, and Titania. J. Colloid Interface Sci..

