# Supplementary material for: Sol–Gel Derived Alumina Particles for the Reinforcement of Copper Films on Brass Substrates
Source: Gels. 2024 Oct 11;10(10):648. doi: 10.3390/gels10100648 (PMC11506922; doi:10.3390/gels10100648)
Supplement: Supplementary file 1 [file gels-10-00648-s001.zip › gels-3244138-supplementary.pdf]

Supplementary Material

1. Characterization methods

A.1. Microstructural and chemical investigations of sol–gel-synthesized alumina (α-Al_2O_3) particles by field emission scanning electron microscope (FE-SEM) with energy-dispersive spectroscopy (EDS) and mapping detection, transmission electron microscope (TEM), and X-ray diffraction (XRD) analysis

A.1.1. FE/SEM-EDS analysis of alumina powder and films

The size, microstructure, and distribution of alumina submicrometre particles obtained from sol–gels were characterized by field emission scanning electron microscope (FE-SEM) (model FE-SEM Mira3 Tescan, Oxford, UK) at 20 kV. The sample was sputtered with a thin layer of Au prior to imaging. EDS analysis was conducted using an INCAx-act LN2-free Analytical Silicon Drift Detector (Oxford Instruments, Oxford, UK), with the PentaFET® Precision and Aztec 4.3 software package (Oxford Instruments, Oxford, UK), connected to the TESCAN Mira3 XMU, Oxford, UK [1].

The distribution of alumina powder was carried out using Image-Pro Plus 6.0 software More than 30 randomly formed different shapes of alumina particles as well as their agglomerates were analyzed.

This FE-SEM was also utilized to investigate the structural features of the pure copper films (Cu) and their composite contra pair (MMC films) with embedded alumina reinforcement in the soft Cu matrix Cu-Al_2O_3. The evaporation with gold was not necessary; the samples of films are conductive. Figure S1a shows the surface morphologies of the MMC Cu-Al_2O_3 film co-electrodeposited from the ABSE-Al-3% electrolyte and MMC Cu-Al_2O_3 films co-electrodeposited from the ABSE-Al-5% electrolyte (S1b).

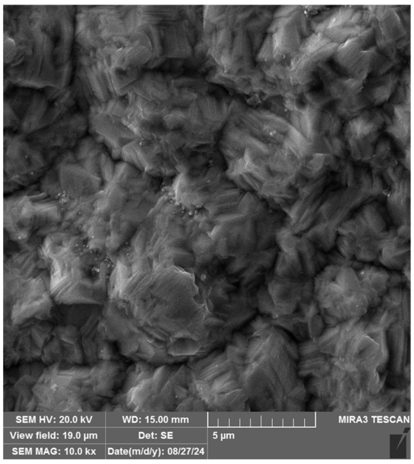

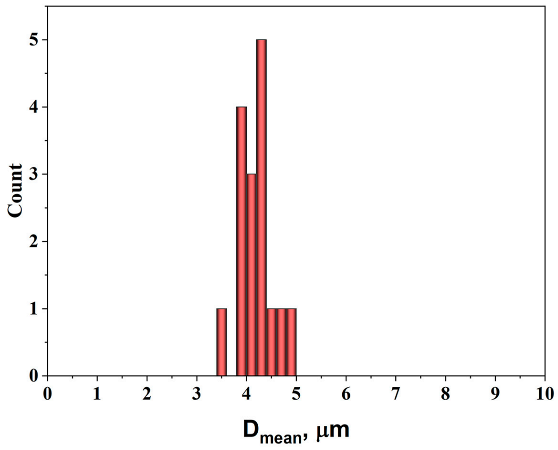

(a)

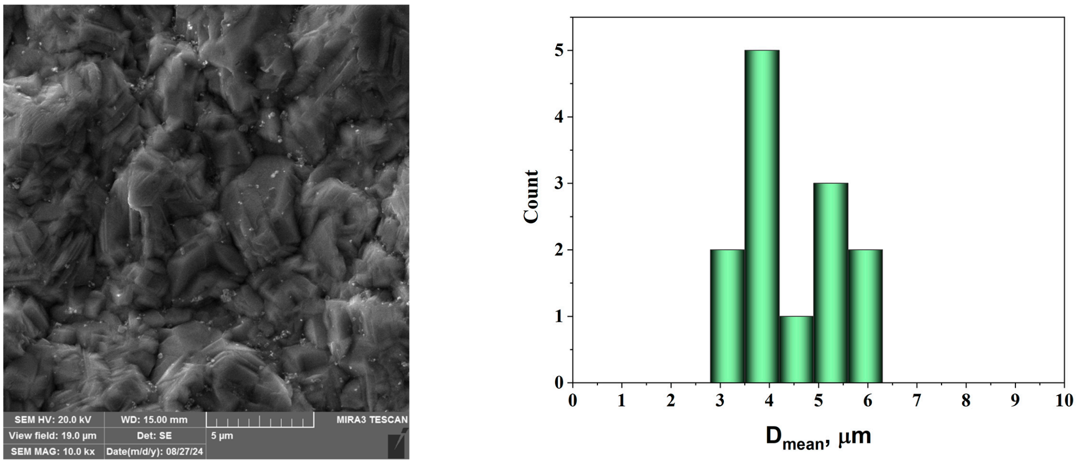

(**b**)

Figure S1. Surface morphologies and histogram analyses of electrodeposited MMC Cu-Al_2O_3 films co-electrodeposited on the brass substrate with a constant thickness of the films (22 μm) and with different concentrations of alumina particles in an ABSE: (a) 3.0 wt. % and (b) 5.0 wt. %. The magnification was × 10 000 for all pictures. The co-electrodeposition mode was DC with a current density of 50 mA·cm^{-2}

Figure S2 shows the mapping analysis of the MMC of Cu-Al_2O_3 films obtained with 1.0 wt.% amounts of the alumina particles (Figure S2a) and with 3.0 wt. % of the alumina particles in an ABSE (Figure S2b).

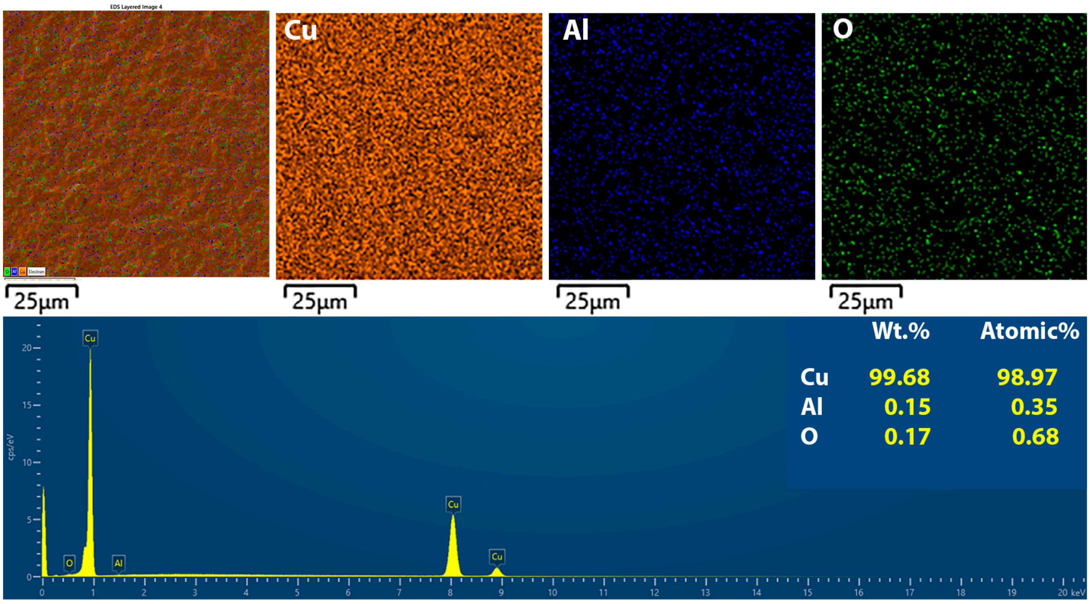

(**a**)

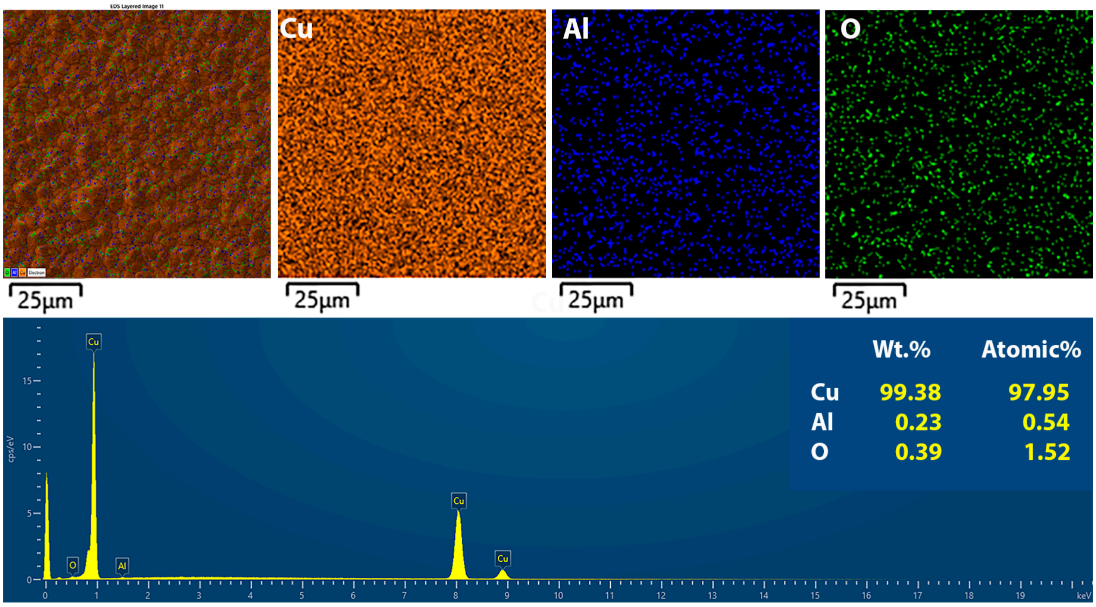

(**b**)

Figure S2. Element mapping of the MMC Cu-Al_2O_3 films co-electrodeposited on the brass substrate with a constant thickness of the films (22 μm) and various concentrations of alumina particles in an ABSE: (**a**) 1.0 wt. % and (**b**) 3.0 wt. %. The film thickness was 22 μm.

A.1.2. Microstructural investigation of sol–gel-synthesized alumina (α-Al_2O_3) particles with transmission electron microscope (TEM)

A transmission electron microscope (TEM), model JEM-1400, JEOL Ltd., Peabody, MA, USA, with an accelerating voltage of 120 kV was used for the investigation of the microstructural analysis of formed agglomerates and particle distribution of Al_2O_3 particles.

A.1.3. The X-ray diffraction (XRD) analysis of alumina powder

An X-ray diffractometer (APD2000, Malvern Panalytical Ltd., Malvern, UK) in Bragg–Brentano geometry was used to undertake X-ray diffraction (XRD) analysis in order to look into the phase structure and crystallinity of the produced alumina nanoparticles. Cu Kα radiation (λ = 1.5418 Å) with a 2θ angle range of 10–80° with a step of 0.02° and a speed of 2°/min was used in the XRD study. The voltage on the X-ray tube was 40 kV, and the current was 30 mA. The mean crystallite size of alumina particles was estimated using the Debye–Scherrer formula (S1) [2]:

$$D = \frac{K \cdot \lambda}{B \cdot \cos\theta} \quad \text{(S1)}$$

where K and λ, respectively, denote the grain figure factor (K = 0.9) and the X-ray's wavelength (λ=0.154 nm), whereas B and θ, respectively, refer to a complete width standard with half the maximum XRD and the Bragg angle. The lattice's constant as well as the average crystallite size values were obtained by applying the pseudo-Voigt [3] profile.

A.2. Characterization of substrate (brass cathode without films)

A.2.1. Topography and roughness analysis by atomic force microscope (AFM)

The three-dimensional (3D) topography of the brass substrate prior to electrodeposition and co-electrodeposition processes was obtained by an atomic force microscope (AFM) (model Auto Probe CP Research; TM Microscopes–Veeco Instruments, Santa Barbara, CA, USA) in the tapping mode. All measurements were conducted with 256 points per line and 256 lines in total. The surface roughness parameters—the root mean square (S_q) and an arithmetic average of the absolute (S_a) roughness of the brass after mechanical polishing and chemical etching in a mixture of three acids—were determined using the accompanying AFM software from the mean image data plane, using SPLab (SPMLab NT Ver. 6.0.2., Veeco Instruments, Santa Barbara, CA, USA) [4]. The scan size area was 50 × 50 μm^2.

This AFM device was utilized to investigate the roughness analyses of the pure copper films (Cu) and their composite contra pair with embedded alumina reinforcement in the soft Cu matrix Cu/Al_2O_3.

A.2.2. Microhardness analysis by Vickers microindentation

After mechanical characterization (microhardness) of the brass cathode after chemical etching, electrodeposited Cu and co-electrodeposited Cu-Al_2O_3 films were characterized using the Vickers microhardness tester "Leitz Kleinert Prufer DURIMET I" (Leitz, Oberkochen, Germany).

When characterizing the microhardness of the films deposited to the bulk substrate form, we recommend the following values:

1) The microhardness for all systems (film + substrate), i.e., a composite microhardness value, H_c;
2) An absolute hardness of the substrate, H_s (i.e., microhardness data obtained on the surface of the cathode without films);
3) An absolute hardness of the films, H_f (i.e., calculated value of film hardness from composite hardness or measured data at low loads).

Microhardness represents one of the most important mechanical features of films, and their hardness can be determined directly using a small indentation load test (case I: thicker film and a slight impact of the substrate hardness) or indirectly using a composite hardness model approach for a determination of an absolute (true) hardness of the film (case II: thin films and a greater contribution to substrate hardness in the value of measured composite hardness) [5].

To apply a composite hardness model, it is necessary to know the absolute hardness of the substrate (cathode without films) before depositing the films.

The Proportional Specimen Resistance model (PSR), formula S2, [6] is suitable for analyzing the variation of substrate microhardness with the applied load. The Proportional Specimen Resistance (PSR) model is used to analyze the behaviour of brass cathodes under indentation testing, particularly focusing on the indentation size effect (ISE). This model considers the elastic parts of the test material and the frictional part at the indenter/material interface during indentation [7]. The H_S value is estimated:

$$\frac{P}{d} = a1 + a2 \cdot d \tag{S2}$$

Here, P is the applied load above which microhardness becomes load-independent and d is the corresponding diagonal length of the indents. A plot of P/d against d will give a linear plot. The slope value multiplied by the geometric constant of the indenter (1.8544) gives the value of the absolute hardness of the substrate (H_S). The parameters a1 and a2 are the constants. The a1 parameter characterizes the load dependence of microhardness and describes the ISE in the PSR model.

The measured composite hardness values of the system are not constant because the hardness is load-dependent. Meyer's power law [8] expresses the variation of the diagonal size in the function of the applied load.

This indenter was also utilized to investigate the mechanical features of the pure copper films (Cu) and their composite contra pair with embedded alumina reinforcement in the soft Cu matrix Cu/Al_2O_3.

For a calculation of the measured (composite) hardness, H_c (in GPa), a range of applied loads (P) between 5 g (0.049 N) and 200 g (1.961 N) and a dwell time of 25 s were applied. The composite microhardness of the films with an included contribution of the substrate (brass) hardness was calculated using standard ASTM E384 and ISO 6507 [9].

The mathematical model, named the Chen–Gao composite hardness model (C–G), was used to eliminate a contribution of substrate hardness and to calculate an intrinsic (true, absolute) hardness, H_f (in GPa), of the films. The theory of the C–G model is described in more studies in the literature [5,10–12]. The equations we used for the calculation are shown below.

According to this model [5,10–12], a composite hardness, H_c, is related with an indentation depth, h, by Eq. (S3):

$$H_c = A + B \cdot \frac{1}{h} + C \cdot \frac{1}{h^{n+1}} \quad \text{(S3)}$$

In Eq. (S3), n is the power index, and it is 1.8 for the "soft film on hard substrate" composite hardness system [11,12], while A, B, and C are parameters obtained by fitting. The dependencies of H_c on h, as well as those obtained by fitting of Eq. (S3), are used in Figures 10a and 10b in the main draft.

Using A, B, and C parameters obtained by the fitting, the absolute hardness of the Cu films (H_f) was calculated according to Eq. (S4):

$$H_f = A \pm \sqrt[n]{\frac{[n \cdot |B|/(n+1)]^{n+1}}{n \cdot |C|}} \quad \text{(S4)}$$

For the "soft film on hard substrate" composite hardness system, a sign "–" is used in Eq. (S4). The calculated values of the film hardness (H_f), as well as parameters A, B, and C obtained by the fitting for the analyzed Cu films and Cu-Al_2O_3 films on brass substrates, are used for adhesion calculation.

Equation (S5) was used to calculate the critical reduced depth, b, parameter representing an estimation of adhesion of any films with a

substrate. The value of the adhesion parameter b is determined from the slope of the linear function fit of the line ΔH = f (δ/d), according to Eq. (S5) [11]:

$$\Delta H = \left[\frac{7 \cdot (n+1) \cdot (H_s - H_f)}{n \cdot b}\right] \cdot \frac{\delta}{d} \quad \text{(S5)}$$

A.2.3. Wettability analyses of free Cu and Cu-Al_2O_3 MMC films—sessile drop method

The wettability of the produced electrodeposits of copper and their composite pair was determined by performing static contact angle measurements with a sessile drop method [13,14]. The volume of 5 μL of water (18 MΩ·cm) was used. Five drops at randomly selected locations were recorded for each deposit. For these purposes, an optical microscope (Delta Optical Smart 5.0 MP Pro, Mińsk Mazowiecki, Poland) fitted with a high-resolution camera was employed [15]. After capturing pictures of the water droplets on a deposit surface (five seconds after the drop was placed), the static contact angles (θ_{WCA}) for water were analyzed using Image-Pro Plus 6.0 software. Ambient conditions (temperature: 25 °C; relative humidity: 50%) were used for the sessile drop method.

References

1. Mladenović, I.O.; Nikolić, N.D.; Jovanov, V.; Radovanović, Ž.M.; Obradov, M.M.; Vasiljević-Radović, D.G.; Vuksanović, M.M. Influence of SiO2 Nanoparticles Extracted from Biomass on the Properties of Electrodeposited Ni Matrix Composite Films on Si(100) Substrate. *Materials (Basel).* **2024**, *17*, 4138, doi:10.3390/ma17164138.
2. Holzwarth, U.; Gibson, N. The Scherrer Equation versus the "Debye-Scherrer Equation." *Nat. Nanotechnol.* **2011**, *6*, 534–534, doi:10.1038/nnano.2011.145.
3. Ganiev, O.K. Application of the Pseudo-Voigt Function to the Analysis of Single-Particle Tunneling Spectra of <math Altimg="si29.Svg" Display="inline" Id="d1e572"> <mi>d</Mi> </Math> -Wave Superconductors. *Phys. C Supercond. its Appl.* **2023**, *610*, 1354280, doi:10.1016/j.physc.2023.1354280.
4. Mladenović, I.O.; Lamovec, J.S.; Vasiljević Radović, D.G.; Vasilić, R.; Radojević, V.J.; Nikolić, N.D. Morphology, Structure and Mechanical Properties of Copper Coatings Electrodeposited by Pulsating Current (PC) Regime on Si(111). *Metals (Basel).* **2020**, *10*, 488, doi:10.3390/met10040488.
5. Mladenović, I.O.; Lamovec, J.S.; Vasiljević Radović, D.G.; Radojević, V.J.; Nikolić, N.D. Mechanical Features of Copper Coatings Electrodeposited by the Pulsating Current (PC) Regime on Si(111) Substrate. *Int. J. Electrochem. Sci.* **2020**, *15*, 12173–12191, doi:10.20964/2020.12.01.
6. Wang, P.; Gao, Y.; Wang, P. A Comparative Study of Indentation Size Effect Models for Different Materials. *Sci. Rep.* **2024**, *14*, 20010, doi:10.1038/s41598-024-71136-5.
7. Petrík, J. On the Load Dependence of Micro-Hardness Measurements: Analysis of Data by Different Models and Evaluation of Measurement Errors. *Arch. Metall. Mater.* **2016**, *61*, 1819–1824, doi:10.1515/amm-2016-0294.
8. Atkinson, M. Calculation of Characteristic Macro-Hardness from Low-Load Indentation Tests. *Mater. Sci. Eng. A* **1995**, *197*, 165–169, doi:10.1016/0921-5093(94)09724-0.
9. Broitman, E. Indentation Hardness Measurements at Macro-, Micro-, and Nanoscale: A Critical Overview. *Tribol. Lett.* **2017**, *65*, 23, doi:10.1007/s11249-016-0805-5.
10. He, J.L. Hardness Measurement of Thin Films: Separation from Composite Hardness. *Appl.*

Phys. Lett. **1996**, *25*, 2002, doi:10.1063/1.117595.
11. Magagnin, L.; Maboudian, R.; Carraro, C. Adhesion Evaluation of Immersion Plating Copper Films on Silicon by Microindentation Measurements. *Thin Solid Films* **2003**, *434*, 100–105, doi:10.1016/S0040-6090(03)00469-3.
12. CHEN, M.; GAO, J. The Adhesion of Copper Films Coated on Silicon and Glass Substrates. *Mod. Phys. Lett. B* **2000**, *14*, 103–108, doi:10.1142/S0217984900000161.
13. Parau, A.C.; Juravlea, G.A.; Raczkowska, J.; Vitelaru, C.; Dinu, M.; Awsiuk, K.; Vranceanu, D.M.; Ungureanu, E.; Cotrut, C.M.; Vladescu, A. Comparison of 316L and Ti6Al4V Biomaterial Coated by ZrCu-Based Thin Films Metallic Glasses: Structure, Morphology, Wettability, Protein Adsorption, Corrosion Resistance, Biomineralization. *Appl. Surf. Sci.* **2023**, *612*, 155800, doi:10.1016/j.apsusc.2022.155800.
14. Drelich, J.; Chibowski, E.; Meng, D.D.; Terpilowski, K. Hydrophilic and Superhydrophilic Surfaces and Materials. *Soft Matter* **2011**, *7*, 9804, doi:10.1039/c1sm05849e.
15. Mladenović, I.O.; Vuksanović, M.M.; Dimitrijević, S.P.; Vasilić, R.; Radojević, V.J.; Vasiljević-Radović, D.G.; Nikolić, N.D. Mechanical Properties of Electrolytically Produced Copper Coatings Reinforced with Pigment Particles. *Metals (Basel).* **2023**, *13*, 1979, doi:10.3390/met13121979.

Disclaimer/Publisher's Note: The statements, opinions and data contained in all publications are solely those of the individual author(s) and contributor(s) and not of MDPI and/or the editor(s). MDPI and/or the editor(s) disclaim responsibility for any injury to people or property resulting from any ideas, methods, instructions or products referred to in the content.
